# Optimization Shape-Memory Situations of a Stimulus Responsive Composite Material

**DOI:** 10.3390/polym13050697

**Published:** 2021-02-25

**Authors:** Wei-Chun Lin, Fang-Yu Fan, Hsing-Chung Cheng, Yi Lin, Yung-Kang Shen, Jing-Shiuan Lai, Liping Wang, Muhammad Ruslin

**Affiliations:** 1 School of Dental Technology, College of Oral Medicine, Taipei Medical University, Taipei 11031, Taiwan; weichun1253@tmu.edu.tw (W.-C.L.); fish884027@tmu.edu.tw (F.-Y.F.); s010326@shsh.tw (J.-S.L.); 2 Department of Dentistry, Taipei Medical University Hospital, Taipei 11031, Taiwan; g4808@tmu.edu.tw; 3 School of Dentistry, College of Oral Medicine, Taipei Medical University, Taipei 11031, Taiwan; 4 Department of Business Administration, Takming University of Science and Technology, Taipei 11401, Taiwan; linyi@takming.edu.tw; 5 School of Pharmacy and Medical Sciences, and UniSA Cancer Research Institute, University of South Australia, Adelaide, SA 5001, Australia; linping.wang@mymail.uni.sa.edu.au; 6 Department of Oral and Maxillofacial Surgery, Faculty of Dentistry, Hasanuddin University, Makassar 90245, Indonesia; m.ruslin@vumc.nl

**Keywords:** 4D printing, stimulus-responsive composite material, heat, deformation and recovery, optimization

## Abstract

In these times of Industrial 4.0 and Health 4.0, people currently want to enhance the ability of science and technology, to focus on patient aspects. However, with intelligent, green energy and biomedicine these days, traditional three-dimensional (3D) printing technology has been unable to meet our needs, so 4D printing has now arisen. In this research, a shape-memory composite material with 3D printing technology was used for 4D printing technology. The authors used fused deposition modeling (FDM) to print a polylactic acid (PLA) strip onto the surface of paper to create a shape-memory composite material, and a stimulus (heat) was used to deform and recover the shape of this material. The deformation angle and recovery angle of the material were studied with various processing parameters (heating temperature, heating time, pitch, and printing speed). This research discusses optimal processing related to shape-memory situations of stimulus-responsive composite materials. The optimal deformation angle (maximum) of the stimulus-responsive composite material was found with a thermal stimulus for an optimal heating temperature of 190 °C, a heating time of 20 s, a pitch of 1.5 mm, and a printing speed of 80 mm/s. The optimal recovery angle (minimum) of this material was found with a thermal stimulus for an optimal heating temperature of 170 °C, a heating time of 90 s, a pitch of 2.0 mm, and a printing speed of 80 mm/s. The most important factor affecting both the deformation and recovery angle of the stimulus-responsive composite material was the heating temperature.

## 1. Introduction

The first, second, third, and fourth industrial revolutions can respectively be summed up in single words: experience, science, networking, and wisdom. Three-dimensional (3D) printing is also referred to as additive manufacturing (AM) and rapid prototyping. 3D printing can be applied in various fields such as healthcare, sensing, robotics, aerospace, biomedicine, consumer products, etc. 3D printing technology has been unable to satisfy people’s current demands. Therefore, 4D printing has now arisen. Tibbits’ team [1] initially introduced 4D printing technology to the world. The “fourth D” of 4D printing represents time, that is, a “time dimension” is added to a printed object. Tibbits’ group [1,2,3,4,5] proposed structures for stylized matter and reconfigurable elements that allow a material to change shape under an external response, and developed a program and calculated the material called a logical substance as a self-assembling stylized and computational physical materials.

Tetsuka and Shin [6] reviewed the recent growth in novel materials and 3D printing techniques to address the demands of conventional 3D printing methodologies, especially in biomedical applications, by examining such things as printing speed, cell growth feasibility, and complex shape achievement. Joshi and Sheikh [7] indicated the 3D printing was revolutionizing the world of manufacturing, even in the highly sophisticated aerospace industry. 3D printing has been able to reduce weight through complex and net shape manufacturing with fewer joints and more-intricate geometry. Dawood et al. [8] revealed that 3D printing made it possible to accurately make one-off, complex geometrical forms from digital data with a variety of materials, locally or in industrial centers. In maxillofacial and implant surgery, it was becoming commonplace and a prerequisite to use anatomical models made by any number of different 3D printing techniques to assist with the planning of complex treatments. Klippstein et al. [9] successfully fabricated functional unmanned aerial vehicle (UAV) porous structures using 3D printing. Experimental and in-flight testing showed that the proposed methodology was efficient for re-designing functional parts for UAV applications which is ideal for lightweight structures such as in aerospace applications.

Miao et al. [10] cultured human bone-derived mesenchymal stem cells using a 3D printing technique to produce a porous scaffold. The scaffold was fixed at a temporary shape at –18 °C but returned to its original shape at room temperature (25 °C). Bakarich et al. [11] used sodium alginate/poly N-isopropyl acrylate ion-coupled entangled gel ink with different concentrations of poly-N-isopropyl acrylate as a 4D printing material. The gel had a reversible length change of 41~49% for a temperature range of 20~60 °C. Lendlein and Langer [12] pointed out that degradable shape-memory polymer sutures were initially loose in their temporary shape, but when the temperature increased above the glass transition temperature (T_g_), the sutures shrank and automatically tightened up or formed a knot. Sun et al. [13] reviewed different stimulus-reflecting shape-memory materials (polymers, ceramics, gels, and complexes), and explored different stimuli, including heat (thermal-reactive material), stress/pressure (mechanical-reactive material), current/voltage (electro-reactive material), magnetic fields (magnetic-reactive material), pH/solutions/humidity (chemical-reactive material), and light (photo-reactive material). Wang [14] found that when polyurethane (PU) was heated to 60 °C, the linear PU polymers elongated by 50%, but then returned to its the original shape after cooling below the glass transition temperature. Sun and Wang [15] developed micro-carrier transport through a small hole (with a thermal/humidity-reactive PU shape-memory polymer), and it was applied as a micro/nano carrier pushed into living cells during surgery or an operation. Serrano et al. [16] developed a shape-memory elastomeric material with hydrogen and carbon percentages and hydrogen-based polyglycol citrate using dodecanediol as a catalyst. The differing shape-memory property was Young’s modulus which changed from 1.28 ± 0.28 MPa at 22 °C (room temperature) to 0.43 ± 0.16 MPa at 37 °C (body temperature). Leng et al. [17] reviewed the shape-memory functions and characteristics of shape-memory polymers, especially for an analysis of externally applied heat, electricity, light, magnetism, solution induction, and other stimuli. Xu et al. [18] reviewed the mechanism of a shape-memory polymer’s effects and characteristics. Liu et al. [19] divided their research into four parts: outputting traditional micro-fabrication with potential topological stylization on a 2D substrate; the formation of a 2D topological substrate by imposing or self-establishing stress; formation of a 3D shape from a 2D substrate by non-planar bending; and applying a hinge to achieve non-planar folding of a 2D substrate. Mitchell et al. [20] reviewed current materials available for 3D printing that have enabled the emergence of 4D printing, which involves a smart material that responds in a programmed way to an external stimulus. Shafranek et al. [21] discussed how AM technologies were expanding the boundaries of materials science and providing an exciting forum for interdisciplinary research. Rayate and Jain [22] reviewed how 4D printing is an extension of 3D printing in which active stimulus-responsive smart materials produce a static structure. The static structure is then converted into another structure when it is exposed to a stimulus (light, heat, pH, water, etc.). Manen et al. [23] presented a novel shape-shifting technique that required only a hobbyist 3D printer and inexpensive off-the-shelf materials. Castro et al. [24] emphasized the development and translation of multifunctional smart materials for 3D/4D bioprinting into tissue engineering (TE) and regenerative medicine (RM). Estelle et al. [25] demonstrated that fused-deposition modeling (FDM) can be applied to fabricate composites with: (1) controlled hyperelastic property gradients and (2) shape-memory behavior. The core-shell configuration could be extended to designs with controlled material composition and relative configuration gradients to realize more-complex deformation and actuation paradigms such as origami structures. Liu et al. [26] studied synergistic effects of 4D printed thermoplastic shape memory polymers (SMPs) and spring steel strips (SSSs) on enhancing shape memory performance. Hybrid composite specimens with the SSS stacked on the top of an SMP layer and larger SSS thicknesses gave rise to larger recovery forces. Noroozi et al. [27] reported on 4D printing used for adaptive metastructures that exploited resonating self-bending elements to filter vibrational and acoustic noises. FDM was implemented to fabricate temperature-responsive SMPs with self-bending features. An FE method (COMSOL) was developed to replicate the shape recovery and self-bending of SMPs. Chow and Ishak [28] described applications of smart polymer nanocomposites (SPNs) in shape memory, self-healing, self-sensing, self-heating, self-cleaning, and energy harvesting. The processing techniques for SPNs, polymerization, melt-compounding, solution mixing, electrospinning, and thermoset-curing are discussed in their review.

Stimulus-responsive materials are categorized into two types: (1) shape-changing materials which change shape whenever a stimulus is employed, and return to their original shape upon removal of the stimulus [13,17,18,19,21,23] and (2) shape-memory materials which require a programming step, during which a sample is first deformed by an external force and then fixed in a temporary shape by a verification process. This temporary shape is metastable and can be retained until an appropriate stimulus is applied to trigger recovery of the original equilibrium shape [13,17,18,19,21,23,25,28]. Subash and Kandasubramanim [29] reported various facts about 4D printing technology owing to the programmability and the use of advanced materials. The pivotal idea of 4D printing is the shape memory effect (SME), conversed in an SMP which superficially reacts to an external inducement which can be electromagnetic radiation, heat, an active source (current and voltage), hydro (moisture, solvents, chemicals, ions, and pH) or a magnetic field. Henriauez et al. [30] indicated that the AM permits the fabrication of fully customized objects with a high level of geometric complexity with reduced fabrication times and costs 4D printing is the fabrication of 3D printed structures that are able to change with time. Smart polymeric materials (hydrogels and active polymers) form the basis of 4D printing. Barrio and Somolinos [31] indicated that the ability of light to trigger specific physicochemical changes in resist, polymers, and other reactive systems with a high degree of spatiotemporal control is pivotal to produce well-defined structures, patterns, and morphologies of interest in areas including microelectronics, photonics, biomedicine, and soft robotics. Those authors provided an overview of various photochemical reactions in polymers, photosensitive materials, and structuring techniques that utilize light. Xie et al. [32] demonstrated a self-powered, flexible multifunctional sensor by combining a polyvinylidene difluoride (PVDF) film with a micro-structured jamming layer. The jamming layers and finger body are fabricated by multi-material 3D printing technology. The sensor acts as an active jamming element to tune the finger stiffness (15~45 N/m) while not affecting the dynamics of the robotic movement. Langford et al. [33] developed a novel concept of deployable scaffolds using a combination of origami and 4D printing technologies. Polylactic acid (PLA) filaments showed constant shape recovery of about 61% regardless of the amount of deformation. Tubular herringbone tessellated origami showed significant deformation capabilities and a high recovery rate of about 96% despite the presence of cracks in the deformed samples. Bodaghi et al. [34] explored reversible energy-absorbing meta-sandwiches manufactured by FDM 4D printing technology. It was found that dual-material auxetic designs are capable of generating a range of non-linear stiffness levels and dissipating energies as per the requirement of energy-absorbing applications. Origami has inspired artists for hundreds of years to transform ordinary sheets of paper into intricate yet beautiful and 2D or 3D geometries. The goal is to transform a flat square sheet of paper into a finished sculpture through folding and sculpting techniques. Callens and Zadpoor [35] reviewed recent work on origami and kirigami to identify techniques that enabled shape shifting of flat sheets into complex geometries. By introducing aspects from differential geometry (the Gaussian curvature), the authors illustrated the fundamental difference between flat sheets and intrinsically curved surfaces, which could such phenomena as gift-wrapping of spheres to wavy edges in plant leaves.

The above articles on 4D printing seldom focus on the research of process optimization. This optimization research can be used as the best way to understand the fast and repeatability of 4D printing research. Optimization research had applied to various research fields. Sreedharan et al. [36] emphasized the effect of processing parameters on the shrinkage rate of an ABS automotive component during injection molding. The melt temperature plays a major role in shrinkage reduction on the molded part. Huang et al. [37] developed an AAO template as a mold in fabricating a nanostructure on a plastic thin film for nanoimprinting. Analytical results demonstrated that imprinting temperature and de-molding temperature were the most important factors in determining the contact angle of molded PLA and PC thin films for nanoimprinting. Chang et al. [38] produced a microneedle array patch consisting of a biodegradable polymer. The Taguchi method was applied to determine the optimum process parameters for microneedle (MN) array fabrication. The most important process parameter is the embossing temperature for PLA MN arrays fabrication by micro-hot-embossing. 

In this research, the authors used paper as a substrate and then added PLA to the paper’s surface to form a stimulus-responsive composite material (PLA/paper). This stimulus-responsive composite material was fabricated by FDM to print a PLA strip onto the paper surface, and its shape was altered by a stimulus (heat). The shape of the composite material (PLA/paper) led to the development of 4D printing technology. Four process parameters (heating temperature, heating time, pitch, and printing speed) were evaluated in this research to produce deformation angles and recovery angles of the stimulus-responsive composite material. This research focused on optimal processing for shape-memory (deformation and recovery) situations of a stimulus-responsive composite material with 4D printing technology.

## 2. Experimental

### 2.1. Fabrication of the Stimulus-Responsive Composite Material

**Figure 1:**
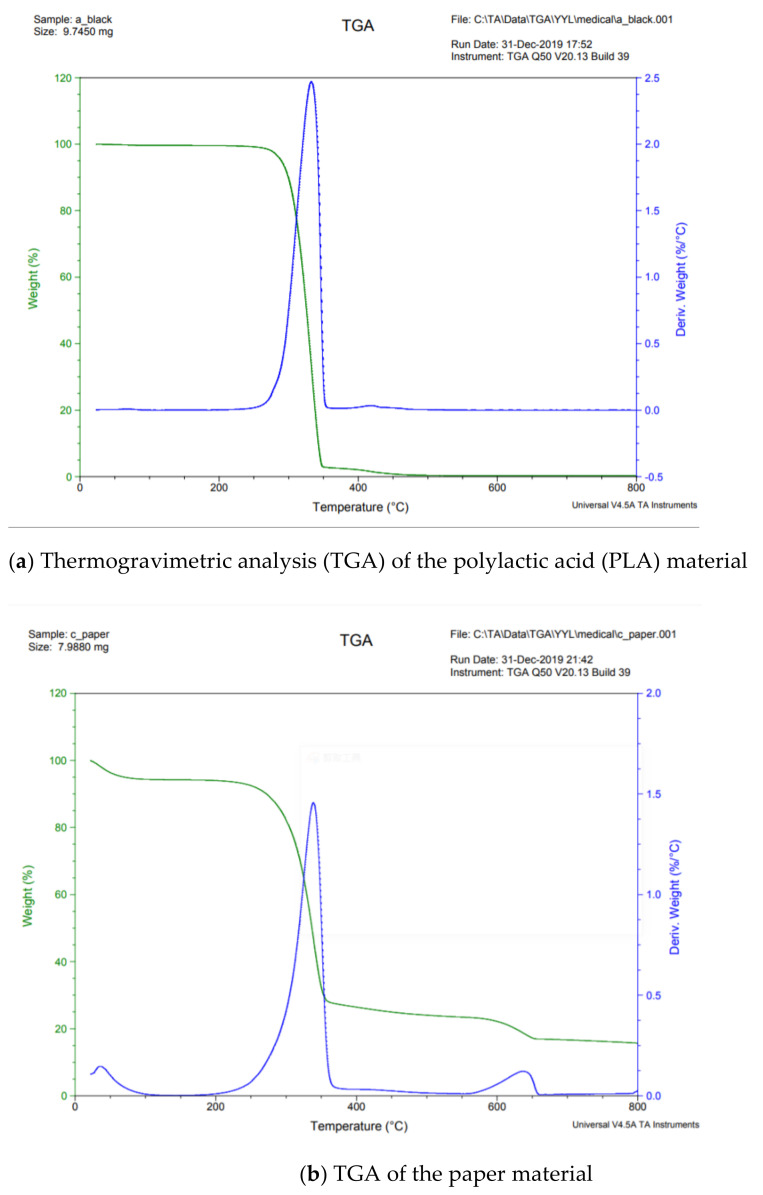

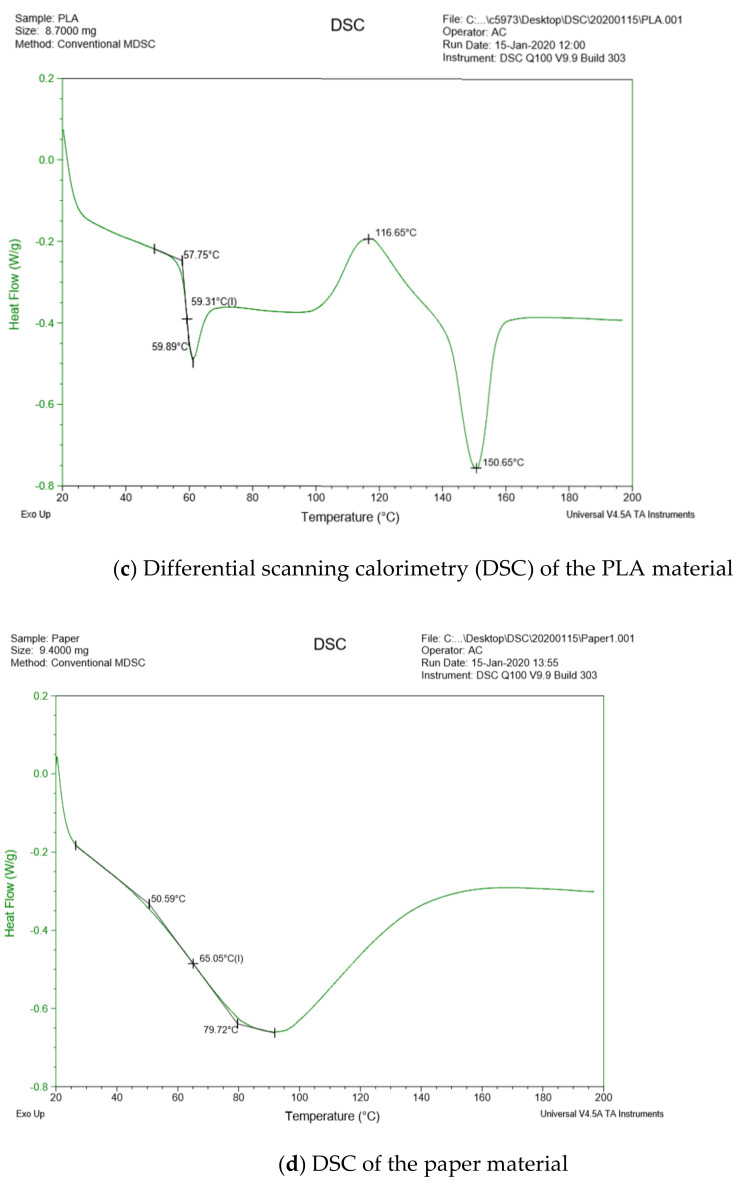
Thermogravimetric analysis (TGA) and differential scanning calorimetric (DSC) analysis of polylatic acid (PLA) and paper.

**Figure 2:**
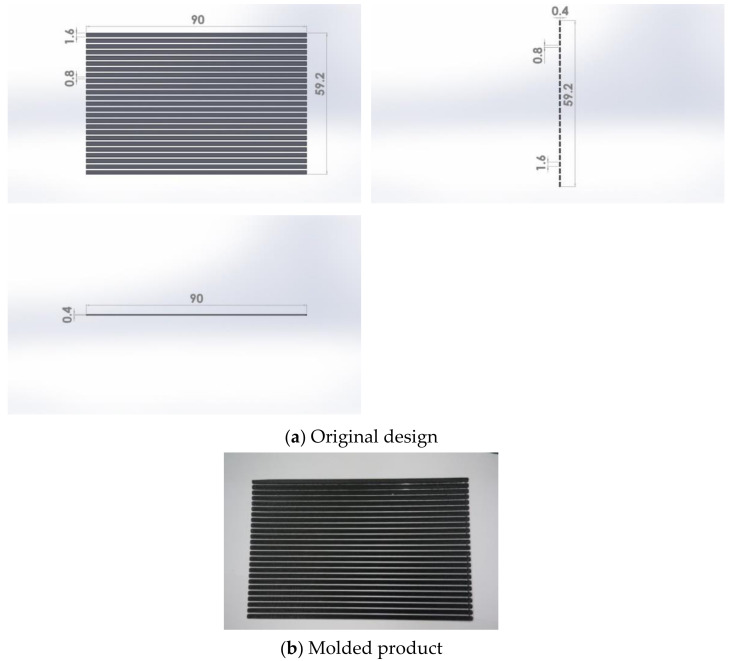
Stimulus-responsive composite material (polylactic acid(PLA)/paper).

PLA was used in a 3D printer, while paper was the substrate. PLA was manufactured by KINGSSEL company, Taiwan. The type of paper was Double A (Double A Company, Taiwan). The mechanical and thermal properties of PLA and paper were reported by from the respective manufactures. In this study, PLA was printed onto the paper’s surface with a 3D printer to produce a stimulus-responsive composite material (PLA/paper). This material was prepared for subsequent 4D printing research. The 3D printer machine used the FDM mode (KINGSSEL 1.0, Taiwan). Its minimum printing size was 400 µm, which was limited by the nozzle size. Its printing speed was 30 mm/s. Thermal properties of the PLA and paper are very important for processing shape-memory stimulus-responsive composite materials. During the printing process, the thickness of the PLA layer was 400 µm. Differential scanning calorimetry (DSC; Q100, TA Instruments, New Castle, DE, USA) and a thermogravimetric analysis (TGA; Q50, TA Instruments, New Castle, DE, USA) were applied to measure the thermal properties of the PLA and paper to double-check the properties reported by the manufacturers (Figure 1). The respective densities of the PLA and paper were 1260 and 10 kg/m^3^. The temperatures of the solid phase of the PLA/paper materials ranged 0~265 and 0~250 °C, and their liquid phases occurred at temperatures above 350 and 365 °C, respectively. Temperatures of the transition phase of the PLA and paper material ranged 265~350 and 250~365 °C, respectively. Results revealed that the glass transition temperature of the PLA material was 59.31 °C, its crystalline temperature was 116.65 °C and its melting temperature was 150.65 °C. The glass transition temperature of the paper material was 65.05 °C. The mechanical and thermal properties of the PLA and paper material are enumerated in Table 1. The original design and molded stimulus-responsive composite material (PLA/paper) are shown in Figure 2. The rectangular size of the paper (white) was 90 × 59.2 × 0.11 mm and that of the strip of PLA material (black) was 90 × 1.6 × 0.4 mm.

### 2.2. Stimulus (Heat)

We attempted to find shape-memory (deformation and recovery) situations of the stimulus-responsive composite material (PLA/paper) under an external stimulus (heat). The hot plate used as the heat source was manufactured by Corning (Corning, NY, USA). Its maximum temperature was 550 °C. The stimulus-responsive composite material (PLA/paper) was placed on the hot plate to determine of the deformation and recovery situations.

**Figure 3:**
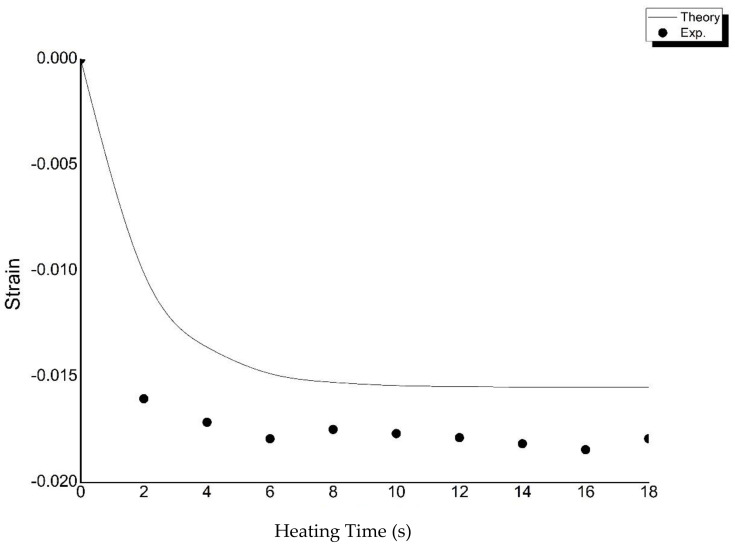
Theoretical and experiment data on the strain-heat time for the printed polylactic acid (PLA) material.

The deformation and recovery circumstances are major points of discussion for stimulus-responsive composite materials used for 4D printing. The authors first wanted to understand the strain diversification of PLA when heat was added. The printed PLA strip (90 × 1.6 × 0.4 mm) was heated on the hot plate to 90 °C. The range of the heating time was 0~18 s. By calculating the ratio of the contraction to the initial length of the strip, the authors obtained the strain of the long strip during the deformation process as shown in Figure 3. As can be seen, the printed strips expanded in the beginning, but the expansion process was very short because the strips were so thin that they reached an equilibrium temperature very quickly. Subsequently, when the temperature exceeded the glass transition temperature (T_g_ = 59.31 °C), the printed PLA strips began to contract, resulting from the release of internal strain. Strain in the experiment was ε = (l_after_ – l_before_)/l_before_, where l_before_ is the original length before adding heat, and l_after_ is the length after adding heat (Figure 3). The moment t_0_ corresponding to T_g_ was the beginning of the release process. A classic Voigt model [39] which consists of a spring and a dashpot can be applied to describe the strain (ε) of the printed PLA strip with an increase in the heating time (t):

(1) $$\varepsilon (t^{\prime })=-\varepsilon_{r}(1-e^{\frac{-t\prime }{\tau_{f}}})$$

where *t*′ = *t − t*_0_. The authors obtained the stored internal strain (ε_r_ = 0.0155), the relaxation time (τ_f_ = 1.89 s), and t_0_ = 0. This research used Equation (1) to fit the experimental data in Figure 3.

As the heating time increased, the strain of the PLA decreased in terms of both the theoretical values and experiment data. This means that PLA became shorter as the heating time increased. The absolute strain value became larger when the heat time was between 10 and 18 s. The absolute value of the strain of the experimental data was larger than that of the theoretical data at the same heating time.

**Figure 4:**
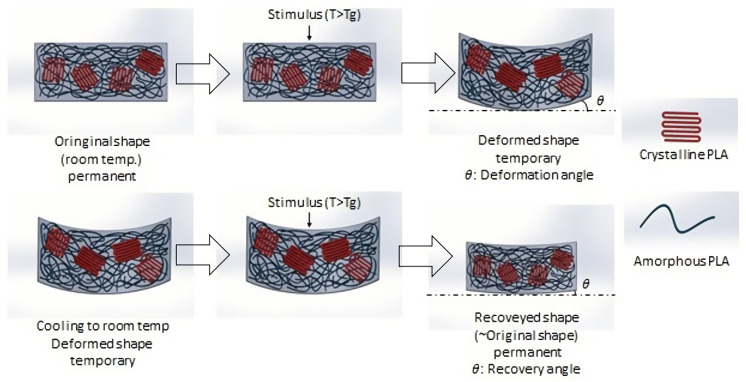
Shape memory mechanism of a stimulus-responsive composite material.

In this study, we discuss the deformation and recovery circumstances of the stimulus-responsive composite material under a thermal stimulus. The SME depends on a material’s ability to recover its original shape when the correct stimulus is applied. The higher-energy barrier between the temporarily deformed shape and the permanent original shape causes the shape effect. Characteristics of the shape memory mechanism for stimulus-responsive composite materials are described in Figure 4. The deformation angle indicates the deformation situation in the experiment. Its value was measured with a protractor. The deformation angle defines the difference between the shape of the object’s deformation curve and its horizontal shape. The recovery angle reveals the recovery situation in the experiment. Its value was also measured with a protractor. The recovery angle defines the difference between the shape of the object’s recovery curve and its horizontal shape. A larger deformation angle, indicates better deformation of the composite material. With a smaller recovery angle, the material can more easily return to its original shape. In this research, the polymer was rubbery and soft above the glass transition temperature. A polymer can easily be deformed. Upon cooling below the glass transition temperature, however, the polymer was glassy and hard. Once heated above the glass transition temperature, the original shape could be recovered by the cross-linking behavior of the polymer, which is an element of stored elastic energy, and it is the driving force for recovering of the shape in the later stage.

Optimization of deformation and recovery of the stimulus-responsive composite material for 4D printing are key points of this study. The authors applied four processing parameters (heating temperature, heating time, pitches of two PLA strips, and printing speed) to optimize the process. The applied heating temperatures of 170, 180, and 190 °C (the value of this parameter were between the glass transition temperature and thermal decomposition temperature of the polymer), the deformation/recovery heating times were 10/30, 15/60, and 20/90 s (this parameter refers to results in Figure 3), values of the pitch were 1.0, 1.5, and 2.0 mm (these values were based on our own laboratory experience), and the applied printing speeds were 40, 80, and 120 mm/s (these values were also based on our own laboratory experience) (Table 2). To identify the relative significance of these four parameters, various experiments were performed for 3^4^ runs. A statistics-based experimental design method, the Taguchi method [40], was utilized to reduce the number of experimental runs. An L_9_ orthogonal array is shown in Table 3. The Taguchi method is a technique for determining optimal combinations of process parameters widely employed in engineering analyses and the world of manufacturing. This method is a very powerful tool for designing and improving high-quality systems, classified into three categories: system design, parameter design, and tolerance design. Of these three design categories, parameter design is the most important with the aim of finding optimal combinations of process conditions to improve performance characteristics. Moreover, the Taguchi method uses two main parameters, i.e., the signal-to-noise (S/N) ratio and orthogonal arrays used in design. This method recommends using a special design of orthogonal arrays to study the entire parameter space without conducting a high number of experiments. The method also integrates orthogonal arrays and the S/N ratio. In the Taguchi method, the S/N ratio is utilized to measure quality characteristics and significant process conditions through an analysis of variance (ANOVA). Deformation and recovery are properties of primary concern when stimulus-responsive composite materials are used for 4D printing. Consequently, variations in the deformation and recovery with different processing parameters and noise were analyzed using the Taguchi method [40]. To maximize the deformation angle of the stimulus-responsive composite material, the following equation, which describes the larger-the-better characteristic, can be used for the analysis.

(2) SN=−10log1n∑i=1n1yi2

where *y_i_* is the measured property (deformation angle of the stimulus-responsive composite material), and *n* is the number of samples in each test trial. To minimize the recovery angle of the stimulus-responsive composite material, the following equation, which depicts a smaller-the-better feature, can be applied for the analysis:

(3) SN=−10log1n∑i=1nyi2

where *y_i_* is the measured property (recovery angle of the stimulus-responsive composite material) and *n* is the number of samples in each test trial.

The measured data were subjected to statistical analyses. For any given experiment, each data point is presented as the mean ± standard deviation (SD) of six individual experiments. The t-test was applied to determine the significance between the deformation angle and recovery angle of two groups. Statistical significance was indicated by * *p* < 0.05, ** *p* < 0.01, and *** *p* < 0.001.

## 3. Results and Discussion

### 3.1. Deformation Angles of the Stimulus-Responsive Composite Materials (PLA/paper) with Different Heating Temperatures and Times

#### 3.1.1. Stimulus of the Heating Temperature

**Figure 5:**
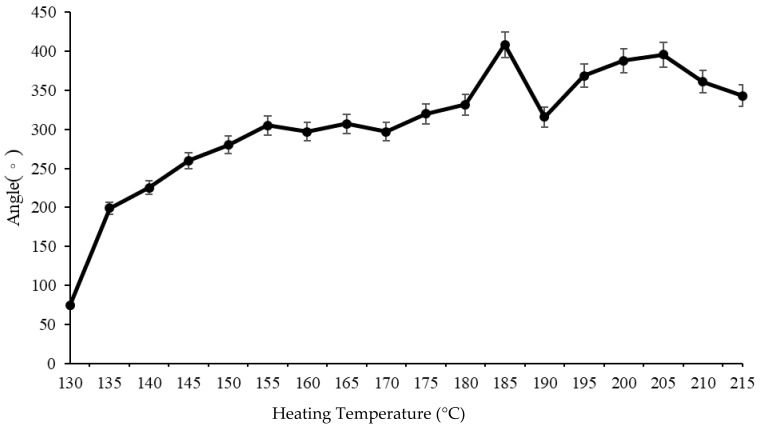
Deformation angles of the stimulus-responsive composite material for at various heating temperatures (with a heating time of 20 s).

There was no reference for the heating temperature used for the stimulus-responsive composite material (PLA/paper) that we could consult. The glass transition temperature of PLA is 59.31 °C, and its melting temperature is 150.65 °C. The temperature of the solid phase of PLA is under 265 °C. The authors first tried to apply 130~215 °C as the heat temperature with the heating time fixed at 20 s to carry out the deformation experiment on the composite material (Figure 5). It was found that with a thermal stimulus of 130~145 °C, the deformation angle of the stimulus-responsive composite material increased very rapidly. At 145~170 °C, the deformation angle of the stimulus-responsive composite material remained nearly constant; and at 170~185 °C, the deformation angle of the stimulus-responsive composite material increased. At 185~190 °C, the deformation angle of the stimulus-responsive composite material tended to decrease; at 190~215 °C, the deformation angle of the stimulus-responsive composite material had an upward trend, and then it began a downward trend. It was found that the stimulus-responsive composite material had the largest deformation angle (408.5°) when the heating temperature was 185 °C. The authors believe that the molecular chain of PLA began to collapse when the heating temperature exceeded 185 °C, so the heating temperature of the composite material was unstable afterwards. Because the deformation angle of the stimulus-responsive composite material decreased at a heating temperature of 205 °C, the authors finally decided to use 170, 180, and 190 °C as the heating temperatures to carry out subsequent experiments. The authors also applied a heating time of 20 s (maximum value) in this study.

#### 3.1.2. Stimulus of the Heating Time

**Figure 6:**
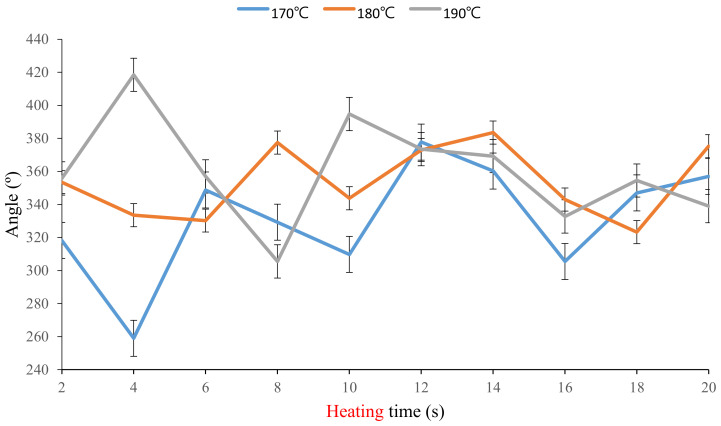
Deformation angles of the stimulus-responsive composite material with various heating temperatures and times.

Figure 6 indicates alterations in the deformation angles of the stimulus-responsive composite material with various heating times at heating temperatures of 170, 180, and 190 °C. Results showed that deformation angles of the composite material at 170, 180, and 190 °C did not greatly differ and they decreased with heating times of 14~16 s. As the heating time increased, the deformation trend of the composite material increased at 170 and 180 °C, but it tended to decrease at 190 °C. The oscillation of the deformation angle of the stimulus-responsive composite material appeared to be alleviated with heating times of 16~20 s. The authors chose heating times of 10, 15, and 20 s for subsequent experiments.

**Figure 7:**
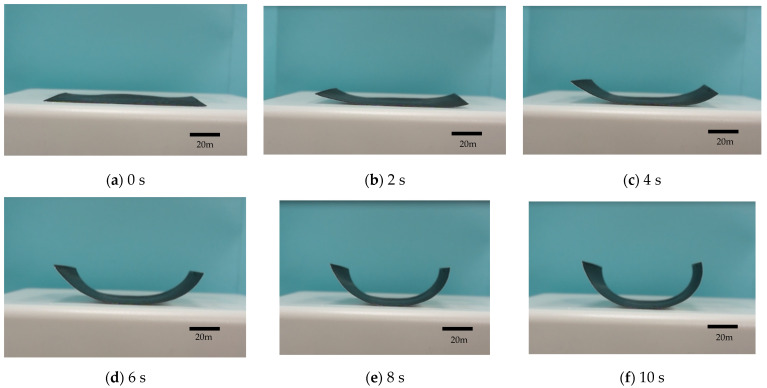

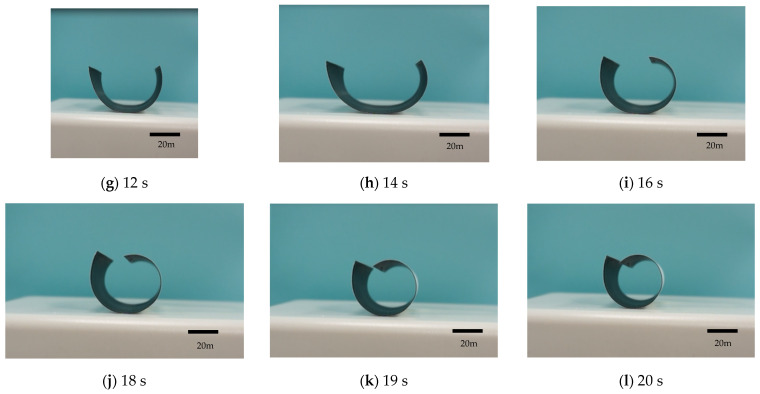
Deformation angles of the stimulus-responsive composite material with various heating times (at a heating temperature of 190 °C and heating time of 0~20 s).

Figure 7 shows the deformation situations of the stimulus-responsive composite material. The heating time ranged from 0 to 20 s in Figure 7a–l. This material had a flat shape. As the heat time increased, the deformation angle of the stimulus-responsive composite material also increased. The shape of the material changed to a hemi-circle type with a heating time of 14 s, and it was a full-circle type with a heating time of 18 s.

### 3.2. Recovery Angle of the Stimulus-Responsive Composite Material with a Thermal Stimulus (Heat)

**Figure 8:**
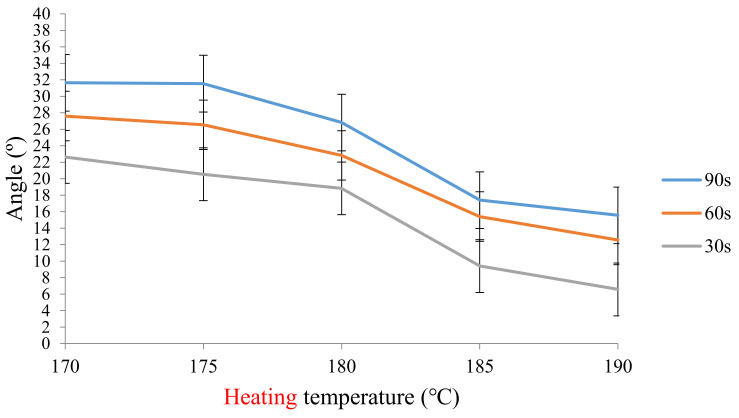
Recovery angle of the stimulus-responsive shape-memory composite material at various heating temperatures (with a heating time of 30, 60, and 90 s).

**Figure 9:**
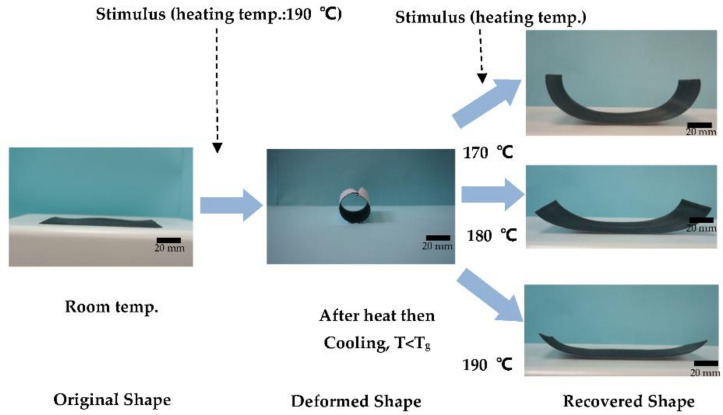
Recovery situations of the stimulus-responsive composite material with heating temperature (190 °C) for the deformation experiment and various heating temperatures for the recovery experiment.

After the stimulus-responsive composite material had deformed at 190 °C for 15 s and it was allowed to cool down to room temperature, it maintained its deformation. The heat source was again turned on to different heating temperatures (170~190 °C), and the recovery angle was observed. Results are presented in Figure 8 and Figure 9. The recovery angle decreased as the heat temperature increased. At a heating temperature of 190 °C, the stimulus-responsive composite material had a minimum recovery angle of 16°. This means that the deformed shape of the stimulus-responsive composite material changed to nearly a flat shape (original shape), indicating good recovery.

### 3.3. Optimization of Shape Changes (Deformation and Recovery) of the Stimulus-Responsive Composite Material for 4D Printing

**Figure 10:**
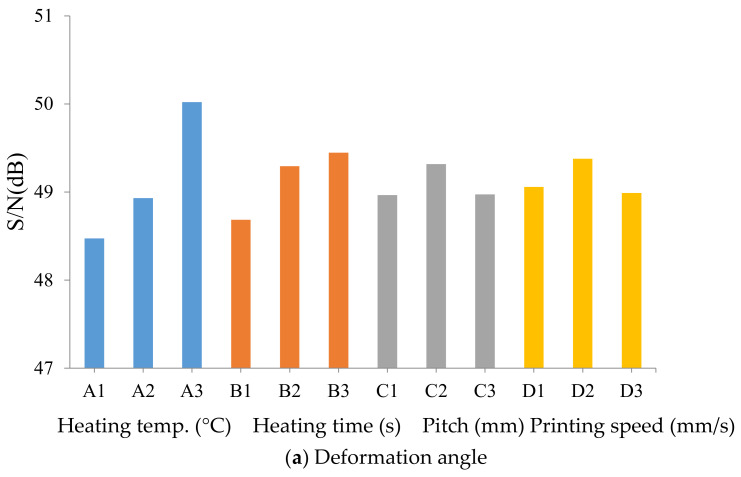

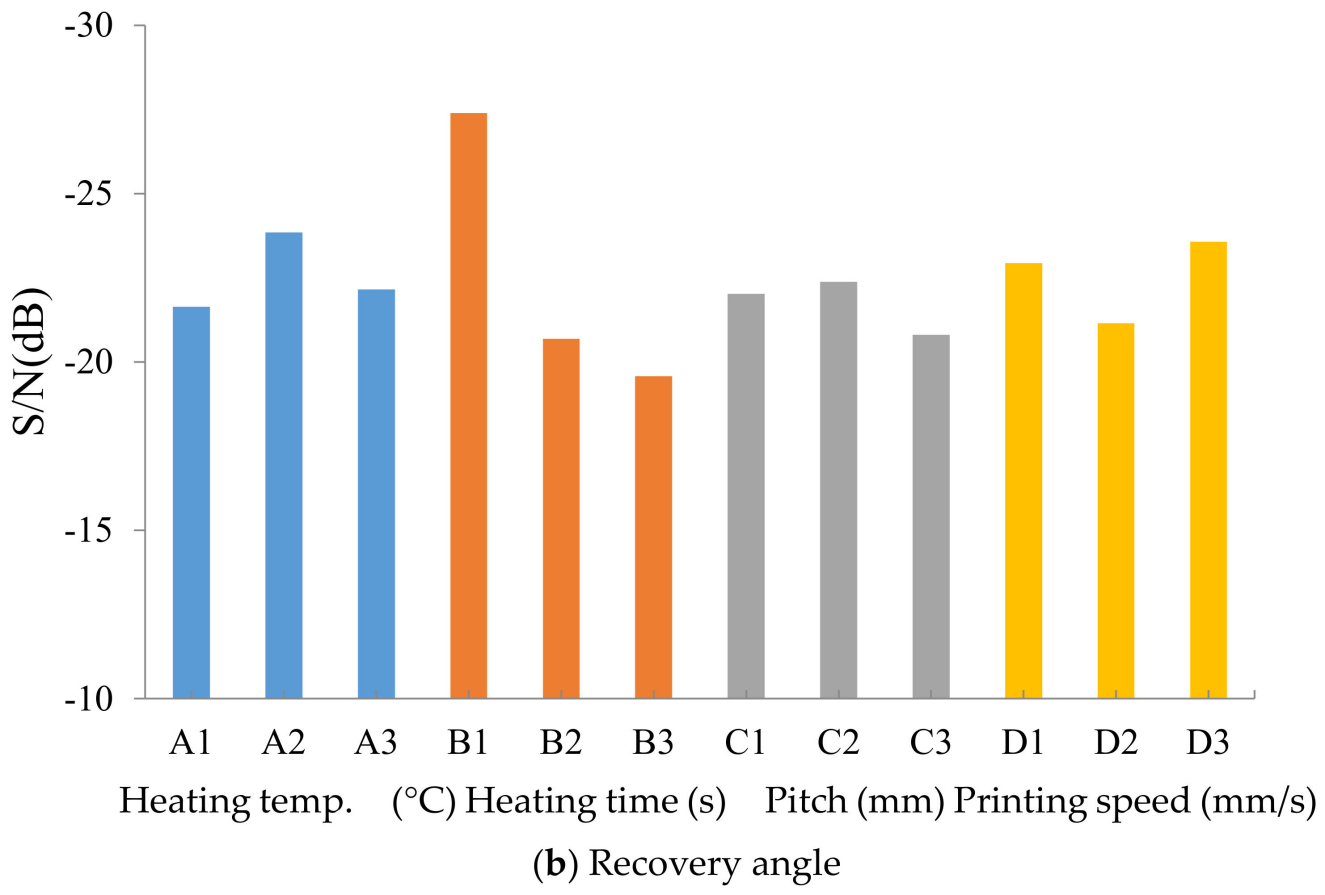
Variations in the signal to nose (S/N) ratio with a factor level for shape changes of the stimulus-responsive composite material.

The optimal deformation angle (maximum value) of the stimulus-responsive composite material with a thermal stimulus and different processing parameters was A3B3C2D2 (Figure 10a). This means that the optimal heat temperature was 190 °C, heating time was 20 s, pitch was 1.5 mm, and printing speed was 80 mm/s. The most important factor affecting the deformation angle of the stimulus-responsive composite material was the heating temperature, followed by the heating time and printing speed; while the pitch was an unimportant factor in the deformation situation. Results also revealed that the optimal recovery angle (the minimum value) of the stimulus-responsive composite material with a thermal stimulus and different processing parameters was A1B3C3D2 (Figure 10b). This means that the optimal heating temperature was 170 °C, heating time was 90 s, pitch was 2.0 mm, and printing speed was 80 mm/s. The most important factor affecting the recovery angle of the stimulus-responsive composite material was the heating temperature, followed by the heating time and pitch, while the printing speed was an unimportant factor in the recovery situation.

**Figure 11:**
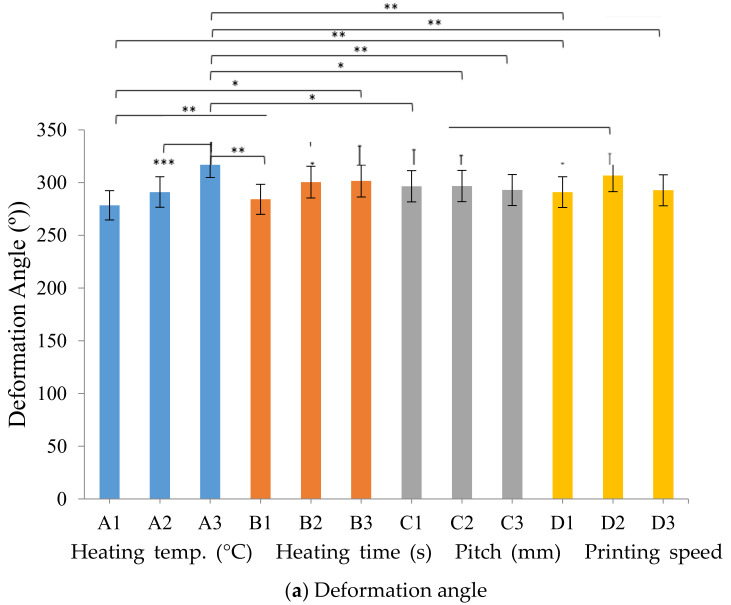

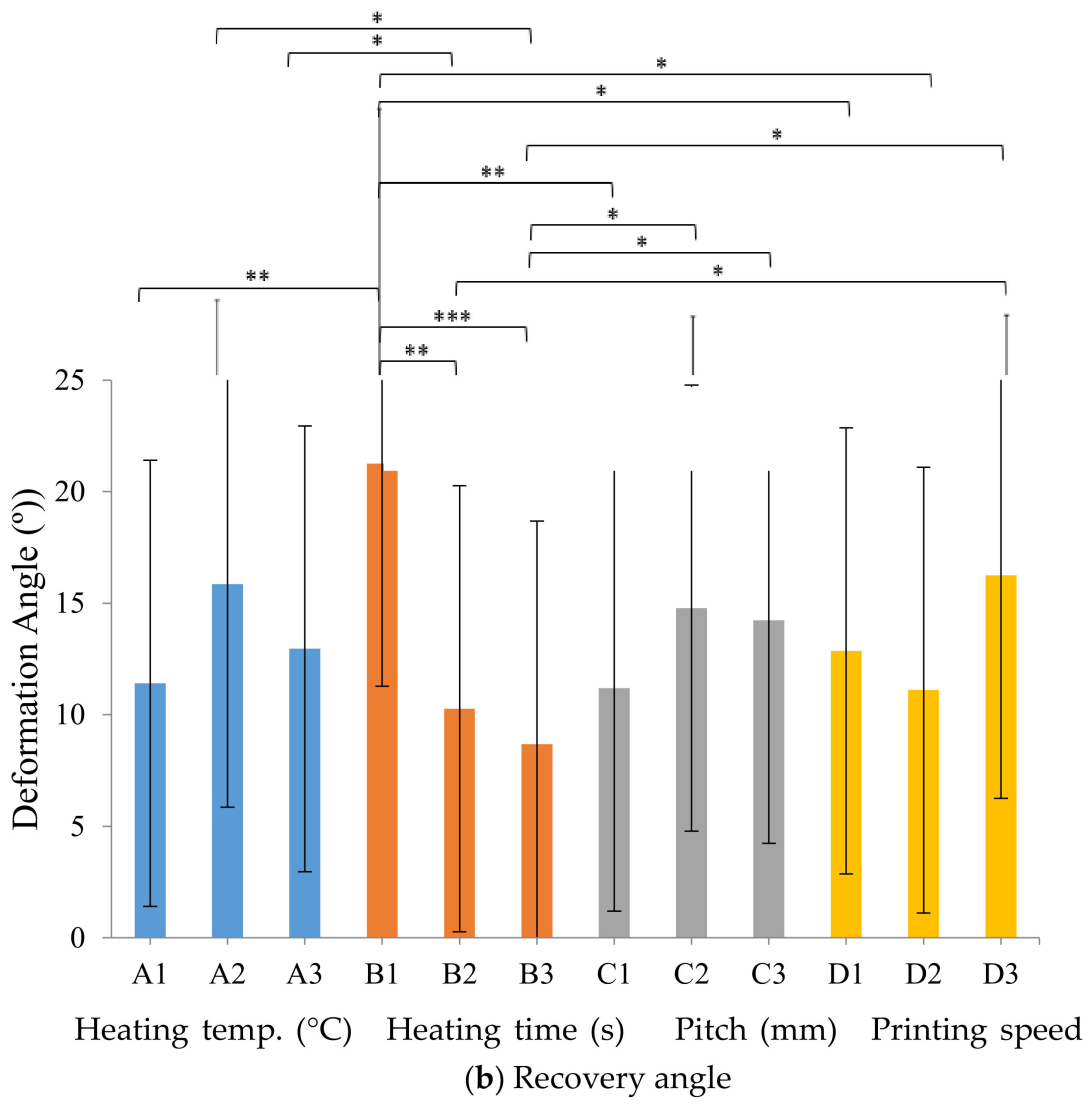
Deformation angle and recovery angle of the stimulus-responsive composite material with various process parameters (values are the mean ± SD of six experiments (*n* = 6), * *p* < 0.05, ** *p* < 0.01, *** *p* < 0.001).

Figure 11 displays the deformation angle and recovery angle of the stimulus-responsive composite material with a thermal stimulus (heat). The deformation angle of this material statistically significantly differed between 170 and 190 °C as the heating temperatures. These results were very similar to the optimal process heating temperature of 190 °C. The recovery angle of the stimulus-responsive composite material statistically significantly differed between heating times of 30 and 90 s. This was similar to the optimal process heat time of 90 s.

### 3.4. Origami Application of the Stimulus-Responsive Composite Material

**Figure 12:**
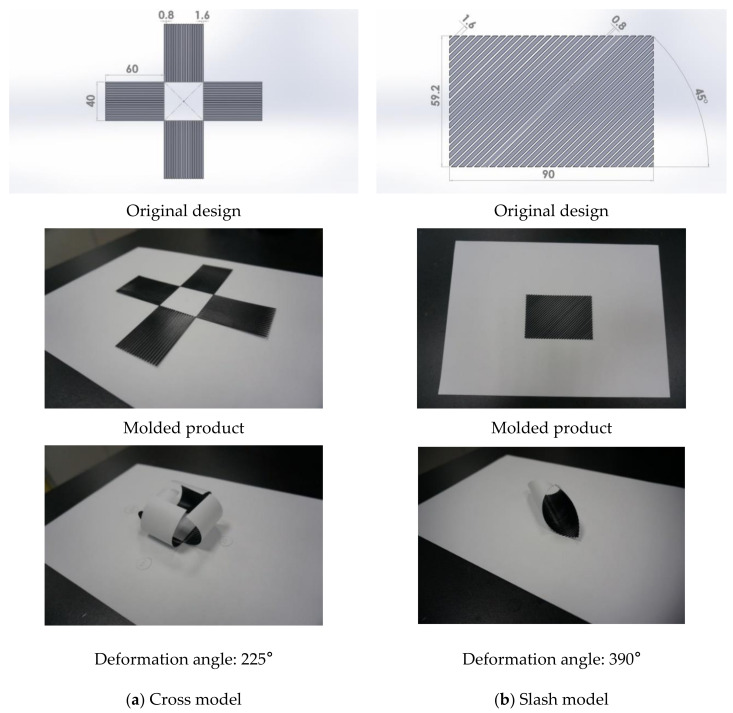
Deformation situations of the stimulus-responsive shape-memory composite material for various models (with a heating temperature of 185 °C, heating time of 16 s).

Origami is an old hand-skilled art. The origami craft can be applied to many designs. The authors wanted to use automated 4D printing technology to replace the hand-skill of origami. Two different models (cross and slash) of the stimulus-responsive composite material were designed; a heating temperature of 185 °C and a heating time of 16 s were applied, and we observed the deformation situation (Figure 12). The four branches of the cross model had a deformation angle of 225° with the stimulus (heat). The deformation angle of the slash modeling was 390° with the stimulus (heat).

## 4. Conclusions

Stimulus-responsive composite material (PLA/paper) was fabricated by fused deposition modeling and subjected to a thermal stimulus for deformation and recovery experiments in 4D printing. The deformation angle of the stimulus-responsive composite material did not change when a higher heat temperature or a longer heat time was applied. The recovery angle of the stimulus-responsive composite material was smaller when a higher heating temperature was applied. At specific heating times (14~16 s), changes in the deformation angle of the stimulus-responsive composite materials under different thermal stimuli (170, 180, and 190 °C) were small and tended to decrease. The recovery angle decreased as the heating temperature increased, revealing a good recovery situation. To sum up, the results show that the most important factor affecting the deformation and recovery angles of the stimulus-responsive composite material was the heating temperature.

## Figures and Tables

**Table 1:** Mechanical and thermal properties of polylactic acid (PLA) and paper.

	PLA	Paper
Young’s modules (E)	2800 MPa	4700 MPa
Density (ρ)	1260 kg/m^3^	10 kg/m^3^
Poisson ratio (υ)	0.33	0.0001
Coefficient of thermal expansion (α)	6*10^−5^/K	2*10^−6^/K
Glass transition temperature (T_g_)	59.31 °C	65.05 °C
Melting temperature (T_m_)	150.65 °C	30 °C

**Table 2:** Level of processing parameters applied for deformation recovery.

	Parameter	A Heating Temperature (°C)	B Heating Time (s)	C Pitch (mm)	D Printing Speed (mm/s)
Experiment					
1		170/170	10/30	1.0/1.0	40/40
2		180/180	15/60	1.5/1.5	80/80
3		190/190	20/90	2.0/2.0	120/120

**Table 3:** The L_9_ orthogonal array used for the main experiment.

	Parameter	A	B	C	D
Experiment					
1		1	1	1	1
2		1	2	2	2
3		1	3	3	3
4		2	1	2	3
5		2	2	3	1
6		2	3	1	2
7		3	1	3	2
8		3	2	1	3
9		3	3	2	1
		(2)			

## Data Availability

The data used to support the findings of this study are included within the article.
